# Lower Serum Iron Level Predicts Postoperative Global Cerebral Edema Following Aneurysmal Subarachnoid Hemorrhage

**DOI:** 10.3390/brainsci13091232

**Published:** 2023-08-23

**Authors:** Haojie Wang, Shufa Zheng, Yibin Zhang, Wenjian Fan, Bingsen Xie, Fuxiang Chen, Yuanxiang Lin, Dezhi Kang

**Affiliations:** 1 Department of Neurosurgery, Neurosurgical Research Institute, First Affiliated Hospital, Fujian Medical University, Fuzhou 350005, China; haojie2016@fjmu.edu.cn (H.W.); zsf2002110@163.com (S.Z.); eabin.z@fjmu.edu.cn (Y.Z.); 711920@fjmu.edu.cn (W.F.); xiebingsen945@sina.com (B.X.); chenfuxiang0404@126.com (F.C.); 2 Department of Neurosurgery, National Regional Medical Center, Binhai Campus of the First Affiliated Hospital, Fujian Medical University, Fuzhou 350212, China; 3 Fujian Provincial Clinical Research Center for Neurological Diseases, The First Affiliated Hospital, Fujian Medical University, No. 22, Chazhong Road, Taijiang District, Fuzhou 350005, China; 4 Fujian Provincial Institutes of Brain Disorders and Brain Sciences, The First Affiliated Hospital, Fujian Medical University, No. 22, Chazhong Road, Taijiang District, Fuzhou 350005, China

**Keywords:** aneurysmal subarachnoid hemorrhage, serum iron, global cerebral edema, prognosis

## Abstract

Background: Iron plays an important role in neuronal injury and edema formation after intracranial hemorrhage. However, the role of serum iron in aneurysmal subarachnoid hemorrhage (aSAH) is yet to be well-established. This study aims to identify whether serum iron could predict postoperative global cerebral edema (GCE) and poor outcome in aSAH. Methods: 847 patients’ aSAH clinical data were retrospectively collected at the First Affiliated Hospital of Fujian Medical University. Data on demographics, clinical characteristics, and laboratory values were collected and analyzed through univariate and multivariate analyses. Propensity score matching (PSM) analysis was performed to balance the baseline differences between the groups. Results: The incidence of high-grade global cerebral edema (H-GCE) following aSAH was 12.99% (110/847). Serum iron levels [odds ratio (OR) = 1.143; 95% confidence interval (CI), (1.097–1.191); *p* < 0.001] were associated with the occurrence of H-GCE following aSAH in the univariate analysis. This association remained statistically significant even after adjusting for other variables in the multivariate model, with serum iron having an OR of 1.091 (95% CI, 1.043–1.141; *p* < 0.001) for GCE. After 1:1 PSM, serum iron levels ≤ 10.7 µmol/L remained a significant independent predictor of GCE (*p* = 0.002). The receiver operating characteristic (ROC) curve analysis determined that a serum iron cut-off value of ≤ 10.7 µmol/L was optimal for predicting H-GCE [Areas under the ROC curves (AUC) = 0.701, 95% CI, (0.669–0.732), *p* < 0.001; sensitivity, 67.27%; specificity, 63.77%] in patients with aSAH. Additionally, a trend was observed in which higher Hunt-Hess grades (HH grade) were associated with lower serum iron levels, and higher modified Fisher grades (mFisher grade) were associated with lower serum iron levels. In addition, the serum iron level was also associated with a 3-month functional neurological outcome (*p* < 0.001). Conclusions: The results of this study indicate that a decreased serum iron level serves as a clinically significant biomarker for the prediction of postoperative GCE and a poor outcome at 3-months in patients with aSAH.

## 1. Introduction

Aneurysmal subarachnoid hemorrhage (aSAH) is a devastating hemorrhagic stroke, with a 30-day fatality of between 33–45% [1]. It has been reported that cerebral edema (CE) occurs in 20–67% of patients following aSAH, which plays an important role in early brain injury within 72 h after SAH [2,3]. Global cerebral edema (GCE) is a special type of CE with an incidence of around 6–8% in the early stages of aSAH [4], which is associated with adverse clinical outcomes and secondary complications, such as cerebral infarction and cerebral hypoperfusion [5,6,7].

GCE is a pathological process that includes microvascular compromise due to ischemia, persistent hypertension, cytotoxic effects of products from lysed blood cells, vasospasm, inflammation, iron-ion disorder, and water dysregulation [2,8,9,10]. Thus, the early identification of patients with high-risk factors of GCE following aSAH is crucial to provide timely and effective interventions to improve the clinical prognosis. Previous studies showed that a few factors could predict the occurrence and progression of GCE following aSAH, such as the Hunt-Hess grade, aneurysm size, loss of consciousness at ictus, and use of vasopressors during hospitalization [11].

Iron is a byproduct of hemoglobin degradation and is implicated in causing arterial structural damage, microcirculatory dysfunction, and neurotoxicity through a series of biochemical reactions in experimental subarachnoid hemorrhage (SAH) [12,13,14]. Neurotoxicity resulting from iron overload may contribute to acute and secondary brain injury, manifesting as delayed brain atrophy, edema formation, cerebral ischemia, and neuronal cell damage, and is associated with early brain injury following aSAH [12,13,15]. Elevated cerebrospinal fluid (CSF) or brain tissue iron levels are associated with cerebral ischemia after SAH [13]. However, the need for invasive sourcing of CSF-iron or brain-iron limits its clinical utility as a biomarker.

Prior clinical studies have illustrated the potential associations of serum iron with iron-mediated neurotoxicity, including acute seizures, hydrocephalus, and postoperative ischemic brain injury following aSAH [16,17,18]. However, few reports have investigated the relationship between serum iron levels and postoperative GCE in aSAH. Therefore, this study aimed to assess the association between serum iron levels and postoperative GCE in patients with aSAH.

## 2. Materials and Methods

### 2.1. Inclusion and Exclusion Criteria

**Figure 1:**
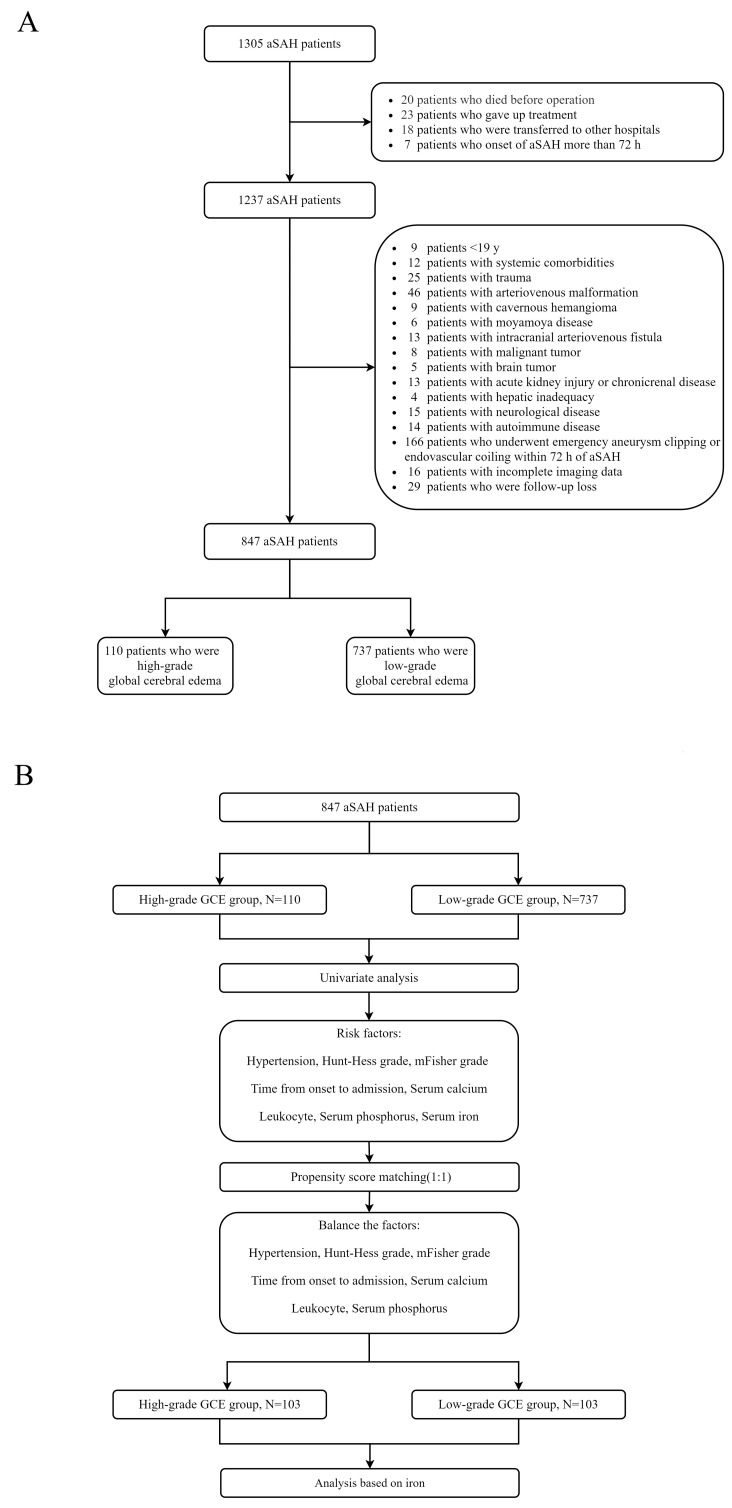
(**A**) Flowchart of patient inclusion; (**B**) Flowchart of propensity score matching.

This study retrospectively collected the clinical data of patients with aSAH at the First Affiliated Hospital of Fujian Medical University. The study was conducted in accordance with the Declaration of Helsinki and approved by the Ethics Committee of the First Affiliated Hospital of Fujian Medical University. Patients diagnosed with aSAH between January 2013 and December 2019 were included in the study. The inclusion criteria were as follows: (1) age ≥ 18 years old; (2) spontaneous SAH diagnosed by computed tomography (CT); (3) presence of intracranial aneurysm confirmed by CT angiography (CTA) or digital subtraction angiography (DSA); (4) admission within 72 h after aSAH; and (5) initial blood sampling for laboratory test within 24 h after admission. The exclusion criteria were as follows: (1) age < 18 years; (2) traumatic subarachnoid hemorrhage; (3) onset of aSAH for more than 72 h; (4) in combination with other cerebrovascular diseases, such as cavernous cerebral malformations, cerebral arteriovenous malformations, moyamoya disease, and intracranial arteriovenous fistula; (5) history of systemic diseases including malignant tumors, autoimmune disease, parathyroid hyperthyroidism, chronic lung disease, chronic heart disease, leukemia, hemolytic anemia, sepsis, uremia, and liver cirrhosis; and (6) missing data. A flowchart of the study is presented in Figure 1A.

### 2.2. Laboratory and Clinical Data

The study collected routine blood tests, such as leukocytes and hemoglobin, as well as routine blood biochemical examinations, including serum potassium, sodium, calcium, iron, and phosphate. Additionally, the study recorded various demographic and medical factors, such as sex, age, medical history (including hypertension, diabetes mellitus, hyperlipidemia, and coronary heart disease), smoking and drinking history, Hunt-Hess grade (HH grade), modified Fisher grade (mFisher grade), aneurysm location, time from onset to admission, and surgical treatment (clipping or coiling). The mFisher grade was categorized as “low grade” for grades 0–2 and “high grade” for grades 3–4, and mFisher grade 3–4 as “high grade”; HH grade I–III as “low grade”, and HH grade IV–V as “high grade” [19,20,21].

**Figure 2:**
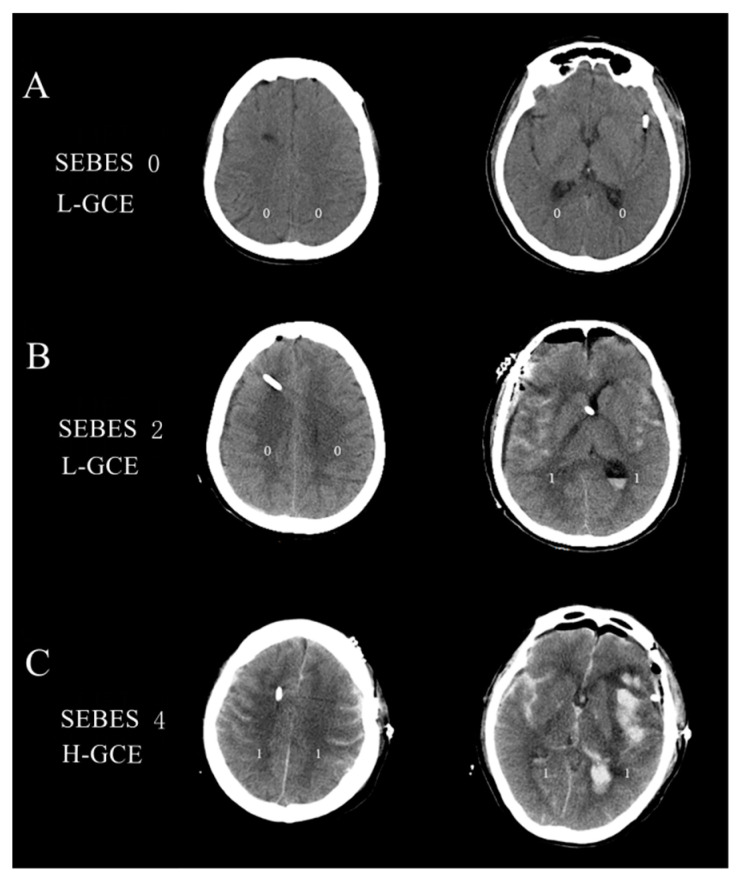
(**A**) Brain CT image of the L-GCE group, with a SEBES score of 0; (**B**) Brain CT image of the L-GCE group, with a SEBES score of 2; (**C**) Brain CT image of the H-GCE group, with a SEBES score of 4.

The subarachnoid hemorrhage early brain edema score (SEBES) grade was used to describe the severity of GCE based on the postoperative brain CT scans [22]. SEBESs with scores 0–2 were defined as low-grade GCE (L-GCE), and scores 3–4 as high-grade GCE (H-GCE) [22]. Figure 2 illustrates the brain CT scans of postoperative L-GCE and H-GCE as measured by the SEBES score. All cerebral CT scans were quantified by two independent neurosurgeons and two neuroradiologists blinded to the clinical data, using SEBES grades ranging between 0 and 4. One point was assigned for: (1) the absence of visible sulci due to effacement of sulci; (2) the absence of visible sulci with disruption of the gray-white matter junction at two predetermined levels in each hemisphere: (a) at the level of the insular cortex showing the thalamus and basal ganglion above the basal cistern and (b) at the level of the centrum semiovale above the level of the lateral ventricle. The presence of sulci was scored as 0, whereas absence was scored as 1. Two slices on both sides resulted in a maximum score of 4.

### 2.3. Clinical Management

All Patients were managed according to the current aSAH guidelines [23]. All patients were administered nimodipine upon admission to prevent vasospasm. During nimodipine administration, blood pressure was consistently monitored and maintained at a systolic range of 110–130 mmHg or the lower limit of the patient’s blood pressure. Fluid therapy was prescribed, and acid-base balance was monitored to prevent hypovolemia and ensure sufficient cerebral perfusion pressure. The treatment of intracranial aneurysms involves either microsurgical clipping or endovascular coiling under general anesthesia using the established techniques. Postoperative management included nimodipine for anti-vasospasm, treatments for the control of CE, blood pressure, nutritional support, and the prevention of complications [24].

### 2.4. Clinical Outcome Assessment

Neurological outcome was followed up until death or three months after discharge. Clinical outcome was evaluated using the modified Rankin scale (mRS). Good clinical outcome was defined as mRS 0–2, while poor clinical outcome was defined as mRS 3–6.

## 3. Statistical Analysis

All statistical analyses were conducted using the SPSS software (version 27.0, IBM SPSS, IBM Corp., Armonk, NY, USA) and Prism 8.3.0 (GraphPad Software, San Diego, CA, USA). The χ2 test or Fisher’s exact test was used to compare other groups for categorical variables. Normally distributed continuous variables are expressed as means ± standard deviation (SD) and analyzed using a 2-sample t-test. Non-normally distributed variables are expressed as median (interquartile range, IQR) using the Mann–Whitney U test or Kruskal–Wallis intergroup differences. Spearman’s correlation analysis was performed to assess the correlation coefficients between the two variables.

All clinical variables associated with H-GCE (*p* < 0.10) in the univariate analysis were incorporated into the multivariate models. The multivariate models were built using logistic regression with the H-GCE status as the dependent variable, which was subsequently implemented to develop a risk model. Receiver operating characteristic (ROC) curve analysis was performed to identify the indicators’ predictive values and calculate the corresponding predictors’ best critical values, sensitivities, and specificities. The Z-test was used to compare the predictive values of different indicators by comparing the areas under the ROC curves (AUC). Propensity-score matching (PSM) was performed to remove imbalances in the basic clinical characteristics between the H-GCE and L-GCE groups (Figure 1B). Conditional probability was estimated using a logistic regression model. The good outcome and poor outcome groups were matched at a ratio of 1:1 using the nearest neighboring matching algorithm.

## 4. Results

### 4.1. Baseline Characteristics

Overall, 847 aSAH patients were enrolled in this study. High-grade SEBES (scores 3–4) was identified in 12.99% of the patients (110/847), and they were categorized into the H-GCE (*n* = 110) and L-GCE groups (n-737). Of the 847 patients, 506 were female and 341 were male. A comparison of the demographic characteristics and clinical data between the two groups is depicted in Table 1.

### 4.2. Risk Factors for H-GCE

**Figure 3:**
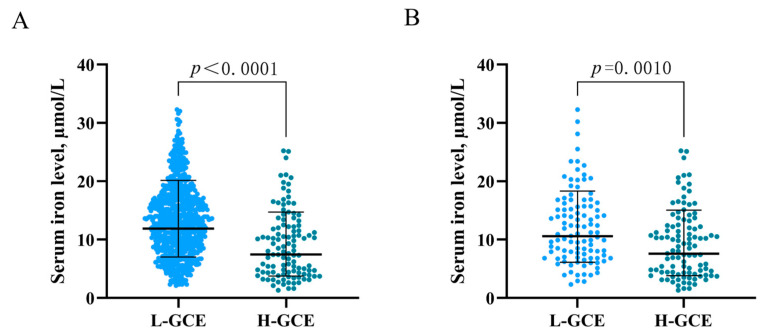
Association of serum iron levels with GCE. (**A**) serum iron levels in patients with L-GCE (*n* = 737) and H-GCE (*n* = 110). (**B**) serum iron levels in patients with L-GCE (*n* = 103) and H-GCE (*n* = 103) after PSM. Mean with standard deviation was shown for all scatter plots in (**A**,**B**).

In the univariable analysis, HH Grades IV–V [OR = 4.808; 95% CI, (3.118–7.413); *p* < 0.001] and mFisher Grade 3–4) [OR = 4.501; 95% CI, (2.966–6.831), *p* < 0.001] were associated with H-GCE. Hypertension [OR = 3.038; 95% CI, (1.941–4.756); *p* < 0.001] was significantly associated with H-GCE. Leukocyte [OR = 0.881; 95% CI, (0.845–0.917); *p* < 0.001], serum calcium [OR = 5.337; 95% CI, (1.341–21.245); *p* = 0.018], serum iron [OR = 1.143; 95% CI, (1.097–1.191); *p* < 0.001], and serum phosphorus [OR = 6.808; 95% CI, (3.293–14.074); *p* < 0.001] levels were associated with the occurrence of H-GCE following aSAH (Table 1 and Table 2). Time from onset to admission [OR = 1.037; 95% CI, (1.002–1.073); *p* = 0.039] also demonstrated a significant association with H-GCE (Table 1). Patients with H-GCE had significantly lower serum iron levels than those with L-HCE (9.186 ± 5.726 µmol/L vs. 13.425 ± 6.139 µmol/L; *p* < 0.001) (Table 1 and Figure 3A).

**Figure 4:**
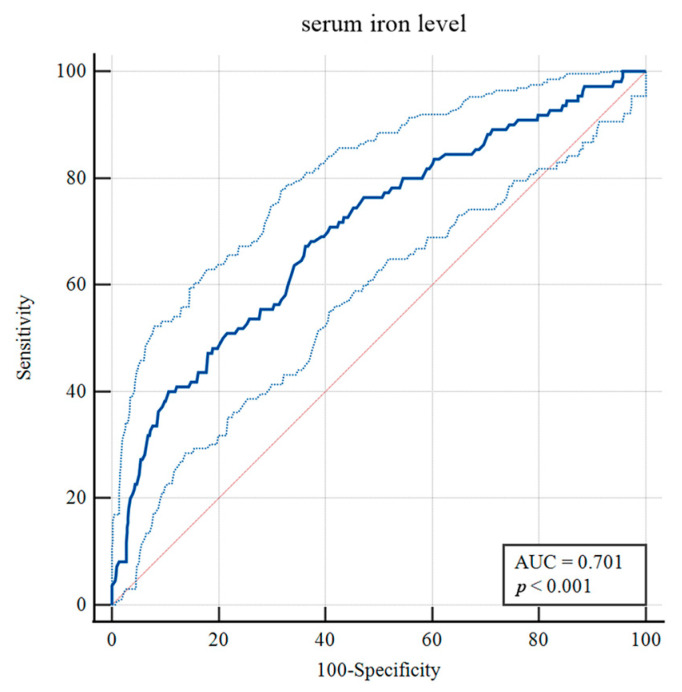
Receiver operating curve analysis of the serum iron levels for predicting H-GCE. (The upper and lower light-blue lines are the 95% confidence intervals).

To identify risk factors for H-GCE, the variables with *p*-values less than 0.10 were included in the multivariable analysis. After adjustment in the multivariate model, hypertension [OR = 2.130, 95% CI, (1.312–3.458), *p* = 0.002], mFisher grade [OR = 2.292, 95% CI, (1.428–3.679), *p* < 0.001], leukocyte [OR = 0.951, 95% CI, (0.908–0.997), *p* = 0.035] and serum iron [OR = 1.091, 95% CI, (1.043–1.141), *p* < 0.001] were still statistically associated with H-GCE (Table 2). The optimal cut-off value of serum iron for predicting H-GCE was ≤ 10.7 µmol/L [AUC = 0.701, 95% CI, (0.669–0.732), *p* < 0.001; sensitivity, 67.27%; specificity, 63.77%] in aSAH patients (Figure 4).

PSM was employed to balance the risk factors in the univariate analysis to further determine the predictive values of serum iron for H-GCE. After 1:1 PSM, 206 patients with aSAH were included in the analyses (Table 3). No significant differences between the H-GCE and L-GCE groups were detected in terms of hypertension, mFisher grade, and leukocyte. A serum iron level ≤ 10.7 µmol/L remained an independent risk factor for H-GCE (*p* = 0.001, Table 3). The matched H-GCE group correlated with significantly lower serum iron levels than the matched L-HCE group (9.358 ± 5.761 µmol/L vs. 12.146 ± 6.258 µmol/L; *p* < 0.001) (Table 3 and Figure 3B).

### 4.3. Association of Serum Iron with HHgrade and mFisher Grade

**Figure 5:**
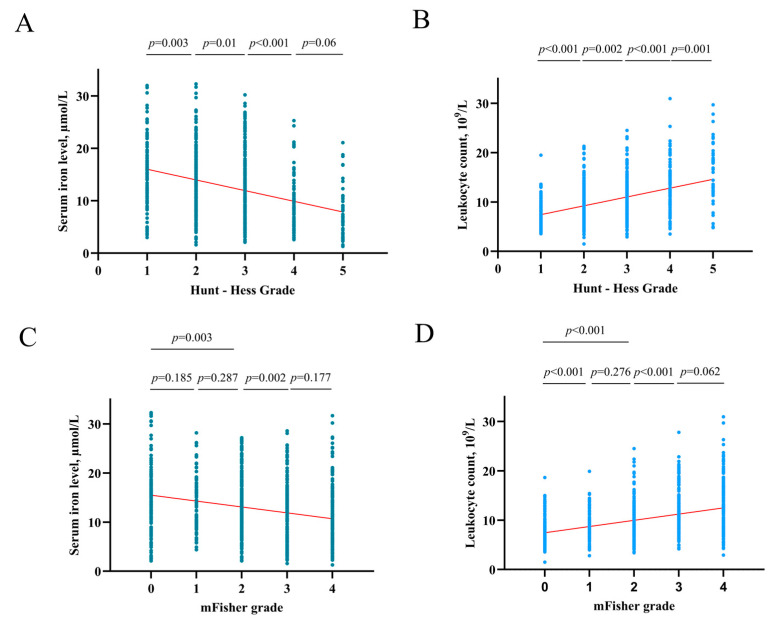
(**A**) The correlation of serum iron levels with Hunt-Hess grade. (**B**) The correlation of leukocyte counts with Hunt-Hess grade. (**C**) The correlation of serum iron levels with mFisher grade. (**D**) The correlation of leukocyte counts with mFisher grade.

**Figure 6:**
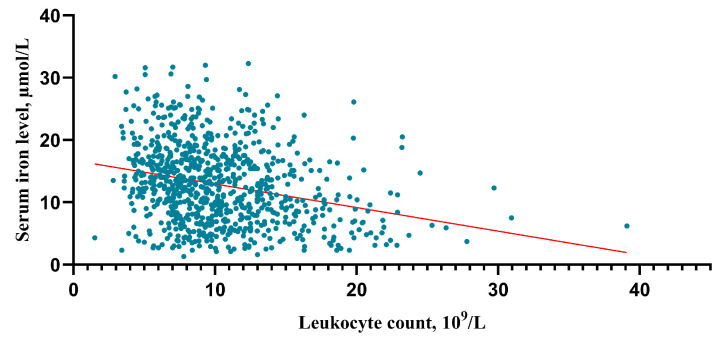
Correlation analysis between serum irons and leukocytes. The correlation was performed using a Spearman’s correlation analysis.

After an in-depth analysis of the upper data, there was a tendency that the higher the HH grade, the lower the serum iron level (HH I grade group vs. II, *p* = 0.003; II vs. III, *p* = 0.01; III vs. IV *p* < 0.001; IV vs. V *p* = 0.06) (Figure 5A). The higher the HH grade, the higher the leukocyte counts (HH I grade group vs. II, *p* < 0.001; II vs. III, *p* = 0.002; III vs. IV *p* < 0.001; IV vs. V *p* = 0.001) (Figure 5B). And this study found that the higher the mFisher grade, the lower the serum iron level (mFisher 0 grade group vs. 1, *p* = 0.185; 1 vs. 2, *p* = 0.287; 0 vs. 2, *p* = 0.003; 2 vs. 3 *p* = 0.002; 3 vs. 4 *p* = 0.177) (Figure 5C). In addition, the higher the mFisher grade, the higher the leukocyte counts (mFisher 0 grade group vs. 1, *p* < 0.001; 1 vs. 2, *p* = 0.276; 0 vs. 2, *p* < 0.001; 2 vs. 3 *p* < 0.001; 3 vs. 4 *p* = 0.06) (Figure 5D). The Spearman’s correlation analysis showed a statistically significant negative association between serum iron and leukocyte (R = −0.271, *p* < 0.001) (Figure 6).

### 4.4. Association of Serum Iron with Poor 3-Month Functional Outcome

**Figure 7:**
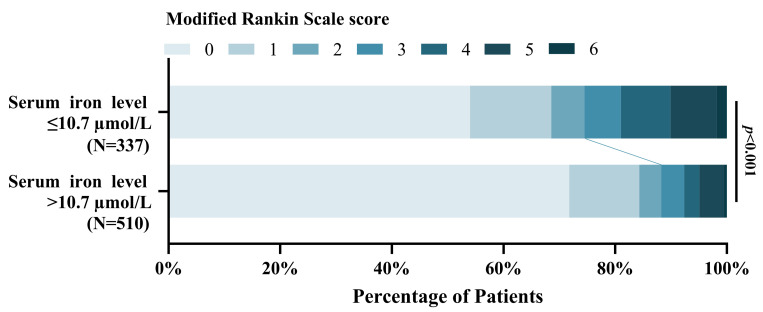
Distribution of 3-month mRS scores of the patients with lower serum iron level ≤ 10.7 µmol/L and > 10.7 µmol/L.

This study found that the patients with lower serum iron levels were significantly associated with poor functional outcomes at 3-months compared to higher serum iron patients [OR = 0.901, 95% CI, (0.871–0.932), *p* < 0.001]. Figure 7 shows the distribution of the mRS scores for the lower serum iron patients and higher serum iron patients [mRS,0–2 (251 vs. 450); mRS,3–6 (86 vs. 60)].

## 5. Discussion

The present study examined the association between serum iron and GCE employing the PSM following aSAH. The cardinal findings of this study are that: (1) a lower serum iron level (10.7 ≤ µmol/L) was independently associated with postoperative H-GCE in patients with aSAH; (2) the HH grade, hypertension, mFisher grade, and leukocyte were statistically associated with GCE; (3) a trend was observed where higher HH grades were associated with lower serum iron levels and higher mFisher grades were associated with lower serum iron levels; (4) a statistically significant negative association was found between serum iron and leukocyte; (5) In line with prior studies [16,17], lower serum iron was remarkably correlated with poor prognosis at 3 months following aSAH. These findings, together with the previous reports [16,17,25], imply that decreased iron concentrations in the peripheral blood, already in the acute phase after SAH, are associated with the mechanisms of secondary brain injury. Interestingly, the present study balanced the reported risk factors for GCE following aSAH—including the HH grade, mFisher grade, hypertension, and leukocytes [2,6,13]—and found that lower serum iron levels remained an independent predictor of postoperative H-GCE after a 1:1 PSM. The matched H-GCE group had significantly lower serum iron levels than the matched L-GCE group. The current study is the first to document the potential predictive power of a decreased admission serum iron level as an independent risk factor for postoperative H-GCE following aSAH.

CE is a major component of early brain injury and is a common and well-known complication after aSAH, representing an independent risk factor for poor clinical outcomes [2,4,8]. Forecasting the occurrence of GCE is of paramount importance for the management of aSAH [23]. In clinical prospective observational studies, it was found that the Fisher or mFisher grade—an assessment of the severity of hemorrhage in patients with aSAH—is associated with elevated risk factors for CE [4,8,13]. The fresh blood escapes into the subarachnoid space when the aneurysm wall ruptures, spreading it out irregularly. Subsequently, the bleeding penetrates further into the cortex [17,26]. The oozing blood leads to initial brain damage through physical destruction and the mass effect, as well as secondary brain damage, such as cerebral edema and delayed cerebral infarction, by releasing potentially neurotoxic and proinflammatory factors (e.g., hemoglobin, iron, and peroxiredoxin-2) [17].

The bursting of erythrocytes leads to an augmented hemoglobin (Hb) level in the brain [27]. Iron, a by-product of the breakdown of Hb, is also processed in the cerebral cortex. A clinical observational study initiated by Raimund Helbok and colleagues found that iron accumulates in the cerebral white matter of patients with poor-grade aSAH, potentially confirming the hypothesis that iron is an important contributor to acute brain injury after aSAH and is associated with the mechanisms of secondary brain injury [13]. Iron overloads were detectd in the brain three weeks after SAH, and the subsequent elevated iron level persists throughout the one-year follow-up period [13]. Monitoring iron levels through invasive procedures is uncommon in clinical treatment and research.

Unlike the previous research [28,29,30], this study gauged the iron levels from serum samples rather than directly from CSF or brain tissue. The reported literature has demonstrated that aSAH causes a decrease in serum iron [19,25,31]. The present study observed a trend in which higher HH grades were associated with lower serum iron levels, and higher mFisher grades were associated with lower serum iron levels, implying that serum iron is associated with the clinical condition. A combined multivariable and 1:1 PSM revealed that a lower serum iron level upon admission was an independent predictor of postoperative H-GCE following aSAH.

Nonetheless, the exact mechanism behind the correlation between serum iron and postoperative H-GCE remains unknown. Therefore, herein, we elaborate on the potential mechanisms of serum iron and postoperative H-GCE following aSAH. Firstly, neuroinflammation is an important predictor for the development of GCE [2,6,8]. Systemic inflammatory response syndrome is a widespread phenomenon identified as a major contributor to an increased risk of developing H-GCE following aSAH [1,6]. aSAH initiates a significant inflammatory response in the brain and involves the recruitment of inflammatory cells in the peripheral blood [2,6,8]. Elevated levels of interleukin-10 [32], interleukin-6 (IL-6) [33], white blood cell count (WBC) [34], and tumor necrosis factor-α (TNF-α) [6] were observed following aSAH. These inflammatory biomarkers were independently associated with persistent CE. This study confirmed that elevated leukocytes were associated with postoperative H-GCE.

Interestingly, a statistically significant negative association between serum iron and leukocyte was detected. This result substantiates the reported connection between serum iron and inflammation. The reported literature has demonstrated that inflammatory cytokines affect iron regulation and metabolism in neurological disorders, suggesting that changes in iron metabolism due to cytokine alterations in the early stages lead to a decrease in serum iron levels [35]. Serum iron levels in critically ill patients, including those with SAH, have been observed to decrease in correlation with the inflammatory status of the patient and the duration of their stay in an intensive care unit [31]. The results of the present study indicate that aSAH patients who have experienced H-GCE present with lower serum iron levels, implying a connection between iron metabolism, inflammation, and postoperative H-GCE. On the other hand, inflammatory cytokines, such as TNF-α and IL-6, induce the expression of iron transporter protein receptors and promote iron accumulation in neurons and microglia, accompanied by a decrease in serum iron, leading to the development of GCE [36].

Furthermore, the clinical and experimental data have demonstrated that ischemic injury may be particularly relevant to cerebral edema formation following aSAH and throughout brain injury [2,37]. Iron deficiency leads to a drop in hemoglobin, which immediately follows a lack of oxygen in the blood, resulting in an inadequate supply of oxygen to the brain, ultimately leading to ischemic brain injury and cerebral edema formation. CE worsens with the addition of neuroinflammation [2,38]. Prior studies have demonstrated the association between low serum iron and postoperative delayed cerebral ischemia (DCI). Regrettably, the relationship between DCI and brain edema was not assessed at the time. We plan to explore this further in an upcoming prospective study. In addition, reduced serum iron concentrations have been associated with increased red blood cell oxidative stress [39]. Oxidative stress has been related to various secondary complications, inducing cerebral edema, neuronal apoptosis, and release of reactive oxygen species(ROS) [2,37,40]. Furthermore, both Fe^2+^ and Fe^3+^ are oxidizing, with ROS being produced via the Fenton reaction and the Haber-Weiss cycle [36]. The ROS disrupts the blood-brain barrier (BBB), which results in increased permeability to fluids and solutes. The increased permeability of the BBB contributes to GCE. 

Previous studies have indicated that lower serum iron levels are linked to a worse prognosis three months after a subarachnoid hemorrhage, potentially due to secondary brain injury complications such as GCE, DCI, seizures, and hydrocephalus [16,17,25]. Notably, the optimal cut-off values for forecasting these complications are not consistent. Further studies should be designed to determine the uniform cut-off value for the serum iron level to predict postoperative complications.

This study, which is single-center, observational, and retrospective in design, has certain limitations that prevent us from definitively establishing causal associations. Retrospective studies inevitably suffer from confusion and bias. Additionally, a single measurement may not accurately indicate serum iron levels over time. The optimal accuracy and credibility of this research could have been achieved through continuous dynamic serum iron monitoring, which still needs to be conducted. Furthermore, serum iron levels in peripheral blood may not be a true reflection of the internal environment of the central nervous system and intracerebral iron accumulation following aSAH. Iron accumulates in the cerebral white matter and CSF can provide more accurate and reliable serum data [13]. However, obtaining intracerebral iron accumulation through more invasive and risky procedures potentially restricts its widespread popularity. Moreover, other relevant indicators of iron metabolism were not included in the analysis (relevant data were not collected in this study) [41], which might also affect the accuracy of the findings. The results should be interpreted cautiously and extended to other populations due to differences in metabolic levels, such as iron metabolism between races. Finally, the present study lacked an assessment of CE upon admission. H-GCE is associated with a worse clinical condition on admission, which in turn may indicate a more severe initial injury [42,43]. A worse clinical status is treated with early intervention at our center. Given these limitations, conducting well-designed prospective, multicenter, randomized controlled trials with larger sample sizes in the future is essential for clinicians to treat and manage patients.

## 6. Conclusions

A lower serum iron level upon admission was a clinically significant biomarker for predicting postoperative GCE and a 3-month poor outcome following aSAH. Even though it remains poorly understood, if this represents an epiphenomenon or a pathogenetic mechanism, further basic research studies are necessary.

## Figures and Tables

**Table 1:** Basic clinical characteristics of patients with aneurysmal subarachnoid hemorrhage before propensity score matching.

	Before Propensity-Score Matching		
Characteristics	High-Grade Global Cerebral Edema	Low-Grade Global Cerebral Edema	*p*-Value
	N = 110	N = 737	
Age, mean (SD) y	54.75 (11.874)	54.51 (11.643)	0.841
Gender, female (N, %)	62 (56.36%)	444 (60.24%)	0.439
Medical history (N, %)			
Smoking	18 (16.36%)	131 (17.77%)	0.717
Drinking	14 (12.73%)	76 (10.31%)	0.443
Hypertension	81 (73.64%)	353 (47.90%)	<0.001
diabetes	18 (16.36%)	83 (11.26%)	0.124
Hyperlipidemia	24 (21.82%)	141 (19.13%)	0.507
Coronary heart disease	6 (5.45%)	22 (2.99%)	0.177
Hunt-Hess grade (N, %)			<0.001
Grade I–III	63 (57.27%)	638 (86.57%)	
Grade IV, V	47 (42.73%)	99 (13.43%)	
mFisher grade (N, %)			<0.001
Grade 0–2	54 (49.09%)	599 (81.28%)	
Grade 3–4	56 (50.91%)	138 (18.72%)	
Aneurysm characteristics (N, %)			0.767
Anterior circulation	85 (77.27%)	560(75.98%)	
Posterior circulation	25 (22.73%)	177(24.02%)	
Time from onset to admission, mean (SD) h	11.78 (6.205)	13.01 (5.733)	0.039
Admission laboratory mean (SD)			
Leukocyte,10^9^/L	12.835 (5.297)	9.826 (4.194)	<0.001
Hemoglobin, g/L	131.91 (17.090)	129.24 (17.319)	0.131
Serum potassium, mmol/L	3.851 (0.469)	3.915 (0.464)	0.183
Serum sodium, mmol/L	140.999 (4.284)	140.466 (4.645)	0.258
Serum calcium, mmol/L	2.136 (0.196)	2.171 (0.139)	0.018
Serum iron, µmol/L	9.186 (5.726)	13.425 (6.139)	<0.001
Serum phosphorus, mmol/L	0.842 (0.344)	1.007 (0.347)	<0.001
Treatment (N, %)			0.278
Clipping	69 (62.73%)	422 (57.26%)	
Coiling	41 (37.27%)	315 (42.74%)	

**Table 2:** Risk factors for global cerebral edema of aneurysmal subarachnoid hemorrhage in univariable and multivariable model.

Characteristics	Univariable		Multivariable	
	Odds Ratio (95% CI)	*p* Value	Odds Ratio (95% CI)	*p* Value
Hypertension (N, %)	3.038 (1.941–4.756)	<0.001	2.130 (1.312–3.458)	0.002
Hunt-Hess grade (N, %)	4.808 (3.118–7.413)	<0.001	1.528 (0.900–2.593)	0.116
mFisher grade (N, %)	4.501 (2.966–6.831)	<0.001	2.292 (1.428–3.679)	<0.001
Time from onset to admission, h	0.965 (0.932–0.998)	0.039	1.031 (0.994–1.069)	0.099
Leukocyte,10^9^/L	1.136 (1.090–1.183)	<0.001	0.951 (0.908–0.997)	0.035
Serum calcium, mmol/L	0.187 (0.047–0.746)	0.018	1.410 (0.306–6.499)	0.659
Serum iron, µmol/L	0.875 (0.840–0.911)	<0.001	1.091 (1.043–1.141)	<0.001
Serum phosphorus, mmol/L	0.147 (0.071–0.304)	<0.001	1.860 (0.815–4.241)	0.140

**Table 3:** Basic clinical characteristics of patients with aneurysmal subarachnoid hemorrhage after propensity score matching.

	After Propensity Score Matching		
Characteristics	High-Grade Global Cerebral Edema	Low-Grade Global Cerebral Edema	*p*-Value
	N = 103	N = 103	
Age, mean (SD) y	54.22 (11.893)	55.83 (11.301)	0.323
Gender, female (N, %)	57 (55.34%)	62 (60.19%)	0.481
Medical history (N, %)			
Smoking	17 (16.50%)	15 (14.56%)	0.700
Drinking	14 (13.59%)	8 (7.77%)	0.176
Hypertension	76 (73.79%)	74 (71.84%)	0.754
diabetes	16 (15.53%)	15 (14.56%)	0.845
Hyperlipidemia	22 (21.36%)	16 (15.53%)	0.281
Coronary heart disease	6 (5.83%)	2 (1.94%)	0.149
Hunt-Hess grade (N, %)			0.886
Grade I-III	63 (61.17%)	64 (62.14%)	
Grade IV, V	40 (38.83%)	39 (37.86%)	
mFisher grade (N, %)			1.000
Grade 0–2	54 (52.43%)	54 (52.43%)	
Grade 3–4	49 (47.57%)	49 (47.57%)	
Aneurysm characteristics (N, %)			0.868
Anterior circulation	80 (77.67%)	79 (76.70%)	
Posterior circulation	23 (22.33%)	24 (23.30%)	
Time from onset to admission, mean (SD) h	11.93 (6.214)	10.81 (6.326)	0.199
Admission laboratory mean (SD)			
Leukocyte,10^9^/L	12.724 (5.344)	13.123 (5.777)	0.607
Hemoglobin, g/L	131.21 (17.255)	130.86 (17.794)	0.852
Serum potassium, mmol/L	3.864 (0.456)	3.870 (0.460)	0.920
Serum sodium, mmol/L	141.022 (4.322)	140.571 (4.301)	0.454
Serum calcium, mmol/L	2.144 (0.197)	2.188 (0.119)	0.560
Serum iron, µmol/L	9.358 (5.761)	12.146 (6.258)	0.001
Serum phosphorus, mmol/L	0.868 (0.339)	0.912 (0.281)	0.303
Treatment (N, %)			0.184
Clipping	64 (62.14%)	73 (70.87%)	
Coiling	39 (37.86%)	30 (29.13%)	

## Data Availability

Not applicable.
